# The effects of vascular access types on the survival and quality of life and depression in the incident hemodialysis patients

**DOI:** 10.1080/0886022X.2019.1702558

**Published:** 2019-12-17

**Authors:** Do Hyoung Kim, Ji In Park, Jung Pyo Lee, Yong-Lim Kim, Shin-Wook Kang, Chul Woo Yang, Nam-Ho Kim, Yon Su Kim, Chun Soo Lim

**Affiliations:** a Department of Internal Medicine, Hangang Sacred Heart Hospital, Hallym University Medical Center, Seoul, Korea; b Department of Internal Medicine, Kangwon National University College of Medicine, Chuncheon, Korea; c Clinical Research Center of End Stage Renal Disease in Korea, Daegu, Korea; d Department of Internal Medicine, Seoul National University Boramae Medical Center, Seoul, Korea; e Department of Internal Medicine, Seoul National University College of Medicine, Seoul, Korea; f Department of Internal Medicine, Kyungpook National University School of Medicine, Daegu, Korea; g Department of Internal Medicine, Yonsei University College of Medicine, Seoul, Korea; h Department of Internal Medicine, The Catholic University of Korea College of Medicine, Seoul, Korea; i Department of Internal Medicine, Chonnam National University Medical School, Gwangju, Korea

**Keywords:** Hemodialysis, vascular access, central venous catheter, mortality, quality of life, depression

## Abstract

**Background:**

Although arteriovenous fistula (AVF) is the preferred vascular access for hemodialysis (HD), the association between vascular access types and quality of life is not well-known. We investigated the relationships between HD vascular access types and all-cause mortality, health-related quality of life (HRQOL) and the degree of depression in a large prospective cohort.

**Methods:**

A total of 1461 patients who newly initiated HD were included. The initial vascular access types were classified into AVF, arteriovenous graft (AVG), and central venous catheter (CVC). The primary outcomes were all-cause mortality and HRQOL and depression. The secondary outcome was all-cause hospitalization. Kidney Disease Quality of Life Short Form 36 (KDQOL-36) and Beck’s depression inventory (BDI) scores were measured to assess HRQOL and depression.

**Results:**

Among 1461 patients, we identified 314 patients who started HD via AVF, 76 via AVG, and 1071 via CVC. In the survival analysis, patients with AVF showed significantly better survival compared with patients with other accesses (*p* < .001). The AVF and AVG group had higher KDQOL-36 score and lower BDI score than CVC group at 3 months and 12 months after the initiation of HD. The frequency of hospitalization was higher in patients with AVG compared to those with AVF (AVF 0.7 vs. AVG 1.1 times per year) (*p* = .024).

**Conclusions:**

The patients with AVF had better survival rate and low hospitalization rate, and the patients with AVF or AVG showed both higher HRQOL and lower depression scores than those with CVC.

## Introduction

The types of vascular access were associated with survival of patients with end-stage renal disease (ESRD) undergoing hemodialysis (HD) [1–3]. Among the arteriovenous fistula (AVF), arteriovenous graft (AVG), and central venous catheter (CVC), AVF is the most preferred and recommended vascular access type because of its lower mortality and hospitalization rate. The use of CVC was associated with greater risk of infection which ultimately resulted in higher mortality [4,5]. Despite of this cumulative evidence, over half of incident HD patients inevitably start dialysis via CVC [6,7]. Therefore, we have to continue efforts to prepare permanent vascular access preemptively in impending ESRD patients. In addition, it is necessary to monitor vigilantly the characteristics of patients undergoing HD via CVC and find modifiable factors to be mended.

Besides mortality, another important aspect in choice for vascular access is the quality of life of HD patients. The health-related quality of life (HRQOL) and depression index, Beck’s depression inventory (BDI) are known to be worse in patients with ESRD, and these are probably related to the survival of ESRD patients [8,9]. The type of vascular access seems to be one of the modifiable factors for HRQOL and depression in dialysis population. However, the effects of vascular access type on HRQOL or depression in these patients have not yet been investigated thoroughly. Up to now, only a few studies dealt with the relationship between HRQOL and type of vascular access, and furthermore, the results of studies are not conclusive [10–12]. Furthermore, regarding BDI, there is only one study that showed no relationship between degree of depression and the type of vascular access [10]. These studies even had several limitations in the context of included number of patients and nature of studies. Therefore, a large-scale prospective study is required to determine the relationships between the type of vascular access and HRQOL and depression in dialysis patients.

In this study, we elucidated the characteristics, HRQOL and BDI scores and survival rate according to the initial vascular access type in incident HD patients in a large prospective Korean cohort. Also, the changes in quality of life following 3 months and 12 months after initiation of dialysis were monitored.

## Methods

### Study design and population

This study is a part of prospective cohort study of the Clinical Research Center for End Stage Renal Disease (CRC for ESRD) in Korea. It is a nationwide web-based multi-center prospective cohort study of patients with ESRD, designed to improve survival rate and quality of life and to create effective treatment guidelines (clinicaltrial.gov; NCT00931970). Thirty-one hospitals and clinics in Korea participated, and patients aged 18 years or more with ESRD were enrolled. All patients provided their written consent to participate in this study, which was approved by the institutional review board at each participating center. All clinical investigations were conducted in accordance with the guidelines of the 2008 Declaration of Helsinki.

Over a 7-year period (August 2008 through July 2015), a total of 2154 incident dialysis patients were enrolled in CRC for ESRD. After excluding patients under peritoneal dialysis (*N* = 618) or patients without the information of initial vascular access (*N* = 75), 1461 patients were analyzed in this study.

### Measurements

The types of initial vascular access for HD were classified into AVF, AVG, and CVC. The CVCs were additionally classified as non-tunneled CVC (NTCVC) and tunneled CVC (TCVC). The type of vascular access which was monitored at 3 months or 12 months after HD was considered as a permanent vascular access for each patient.

The following demographic and clinical data were collected and analyzed; sex, time to referral to nephrology, cause of ESRD, body mass index (BMI), systolic and diastolic blood pressure, comorbidities including coronary artery disease (CAD), peripheral vascular disease (PVD), cerebrovascular disease (CVD), congestive heart failure (CHF) and malignancy, and laboratory values including hemoglobin, serum calcium, phosphorus, uric acid, and total cholesterol. In addition, the modified Charlson comorbidity index (MCCI) was calculated for each patient. The MCCI was developed to predict one-year mortality, and it has been validated in ESRD patients [13,14]. Patients were classified as early referral if their first encounter with a nephrologist occurred more than 1 year before initiation of dialysis and received education about dialysis, and all others were classified as late referral, as described previously [15].

### Outcomes

The primary outcomes were all-cause mortality and HRQOL and degree of depression. The secondary outcome was all-cause hospitalization. Kidney Disease Quality of Life Short Form 36 (KDQOL-36) has been used to evaluate the HRQOL of patients with ESRD [16]. We utilized the Korean version of the KDQOL-36, which was recently translated and validated in Korean patients with ESRD [17]. Briefly, this includes 12 items that provide a generic chronic disease core as well as 24 additional kidney disease-targeted items. The 24 additional items focus on particular health-related concerns of individuals with kidney disease (symptom/problem list, 12 items; effects of kidney disease, eight items; burden of disease, four items). The item scores were aggregated without weighting and transformed linearly to a 0–100 possible range, with higher scores indicating better states, which resulted in a total of dimensions. The Korean version of BDI was used to evaluate depression in our patients. The BDI has been validated in various groups of patients and has been used in patients undergoing dialysis to evaluate depression [18]. It consists of 21 self-reported items, and each item is rated on a scale of 0–3, producing a possible score range of 0–63 [19].

Each center recorded information regarding mortality and cause of death on the CRC for ESRD web-based registry. All the medical records of patients who died in hospital registered in CRC for ESRD were checked to confirm the death and mortality date. In case of patient died in other hospitals, information of death was extracted from the Korean National Statistical Office data as of 31 December 2017. Hospitalization was defined as admission for at least 24 h, excluding diagnostic work-ups for transplantation.

### Statistical analyses

Analyses of the differences in baseline characteristics according to the types of vascular access were performed using the *t* test or one-way ANOVA for continuous variables and the *χ*^2^ test for categorical variables. Additionally, in order to compare the quality of life and depression among three groups, univariable and multivariable linear regressions with adjustments for age, sex, type of referral, MCCI and hemoglobin levels as covariates which were selected according to the results of analyses of baseline characteristics were performed. The Kaplan–Meier method was used to compare survival curves, and differences were assessed by means of the log rank test. Statistical analysis was performed using SPSS version 21.0 (SPSS Inc., Chicago, IL). For all analyses, results were considered statistically significant if *p* < .05.

## Results

### Patients’ characteristics according to the type of initial vascular access

CVC was the most common vascular access type and 1071 patients (73.3%) started HD with CVC. AVF and AVG were 21.5% and 5.2%, respectively (Table 1). The age was older in AVG group (61.7 ± 12.5; mean ± SD) compared with AVF (57.3 ± 12.7) and CVC (58.3 ± 14.7) groups. The portion of early referral to nephrologist was higher in the order of AVF, AVG, and CVC. Regarding underlying causes of ESRD, diabetes mellitus (DM) accounted for 69.7% of AVG group, whereas the frequencies of hypertension and glomerulonephritis were relatively higher in AVF and CVC groups. The score of MCCI was significantly higher in patients with AVG which indicate more severe comorbidities than those of patients with AVF and CVC. Especially, prevalence of DM, CVD, PVD and malignancy were significantly higher in AVG group. In laboratory tests, hemoglobin and serum calcium were lower and serum phosphorus was higher in CVC group.

**Table 1.** Baseline characteristics according to the types of initial vascular access.

	AVF *n*= 314 (21.5%) Mean ± SD or *n* (%)	AVG *n*= 76 (5.2%) Mean ± SD or *n* (%)	CVC *n*= 1,071 (73.3%) Mean ± SD or *n* (%)	*p*
Age (years)	57.3 ± 12.7	61.7 ± 12.5	58.3 ± 14.7	.051
Sex, male	207 (66.1%)	43 (56.6%)	652 (60.9%)	.153
Time referral (months)	58.2 ± 123.5	38.0 ± 47.3	40.4 ± 86.5	.015
Type of referral				
Early referral	214 (72.3%)	48 (66.7%)	572 (56.6%)	<.001
Late referral	82 (27.7%)	24 (33.3%)	439 (43.4%)	
Cause of ESRD				
DM	184 (58.8%)	53 (69.7%)	643 (60.0%)	.003
Hypertension	47 (15.0%)	8 (10.5%)	144 (13.4%)	
Glomerulonephritis	44 (14.1%)	6 (7.9%)	131 (12.2%)	
Others	36 (11.5%)	5 (6.6%)	145 (13.5%)	
Unknown	24 (0.6%)	4 (5.3%)	8 (0.7%)	
Score of MCCI	5.4 ± 2.1	6.6 ± 2.6	5.3 ± 2.3	<.001
Comorbidity				
DM	198 (63.5%)	61 (80.3%)	693 (64.8%)	.018
CAD	48 (15.5%)	11 (14.5%)	132 (12.4%)	.342
CHF	31 (10.0%)	12 (15.8%)	113 (10.6%)	.331
CVD	23 (7.5%)	15 (19.7%)	102 (9.6%)	.005
PVD	34 (11.0%)	17 (22.4%)	63 (5.9%)	<.001
Malignancy	24 (7.8%)	16 (21.1%)	67 (6.3%)	<.001
SBP (mmHg)	142.5 ± 21.2	137.7 ± 20.6	145.7 ± 23.9	.003
DBP (mmHg)	77.9 ± 13.3	72.7 ± 12.5	78.3 ± 14.6	.006
BMI (kg/m^2^)	23.5 ± 3.7	23.1 ± 3.4	23.2 ± 3.5	.334
Hemoglobin (g/dL)	9.1 ± 1.5	9.0 ± 1.4	8.7 ± 1.6	<.001
Calcium (mg/dL)	7.9 ± 1.0	8.0 ± 0.9	7.7 ± 1.1	.001
Phosphorus (mg/dL)	5.4 ± 1.7	4.8 ± 1.6	5.6 ± 2.1	.001
Uric acid (mg/dL)	7.9 ± 2.3	7.6 ± 2.5	8.1 ± 2.7	.107
Total cholesterol (mg/dL)	154.0 ± 45.4	137.1 ± 38.4	157.0 ± 50.1	.004
hs-CRP at 0 m	2.4 ± 12.1	6.2 ± 30.1	5.4 ± 18.3	.028

AVF: arteriovenous fistula; SD: standard deviation; AVG: arteriovenous graft; CVC: central venous catheter; ESRD: end stage renal disease; DM: diabetes mellitus; MCCI: modified Charlson comorbidity index; CAD: coronary artery disease; CHF: congestive heart failure; CVD: cerebrovascular disease; PVD: peripheral vascular disease; SBP: systolic blood pressure; DBP: diastolic blood pressure; BMI: body mass index; hs-CRP: highly sensitive C-reactive protein.

The comparisons between TCVC and NTCVC groups are depicted in Table 2. The age was younger in patients with NTCVC compared to patients with TCVC. The referral timing was similar between two groups. The score of MCCI was also similar, but prevalence of CAD, CHF, and CVD was significantly higher in NTCVC group. Laboratory tests showed lower level of hemoglobin and calcium, and higher level of phosphorus and uric acid in patients with NTCVC.

**Table 2.** Comparisons of baseline characteristics between TCVC and NTCVC.

	TCVC *n*= 896 (83.7%) Mean ± SD or *n* (%)	NTCVC *n*= 175 (16.3%) Mean ± SD or *n* (%)	*p*
Age (years)	58.7 ± 14.7	56.3 ± 15.0	.042
Sex, male	536 (59.8%)	116 (66.3%)	.109
Time referral (months)	41.8 ± 93.2	33.4 ± 39.0	.244
Type of referral			
Early referral	472 (56.2%)	100 (58.5%)	.582
Late referral	368 (43.8%)	71 (41.5%)	
Cause of ESRD			
DM	534 (59.6%)	109 (62.3%)	.005
Hypertension	122 (13.6%)	22 (12.6%)	
Glomerulonephritis	99 (11.0%)	32 (18.3%)	
Others	134 (15.0%)	11 (6.3%)	
Unknown	7 (0.8%)	1 (0.6%)	
Score of MCCI	5.3 ± 2.3	5.5 ± 2.4	.312
Comorbidity			
DM	580 (64.9%)	113 (64.6%)	.938
CAD	102 (11.5%)	30 (17.2%)	.035
CHF	68 (7.6%)	45 (25.9%)	<.001
CVD	76 (8.5%)	26 (14.9%)	.009
PVD	49 (5.5%)	14 (8.0%)	.194
Malignancy	56 (6.3%)	11 (6.3%)	.991
SBP (mmHg)	146.1 ± 23.7	143.6 ± 24.7	.214
DBP (mmHg)	78.5 ± 14.8	76.9 ± 14.0	.194
BMI (kg/m^2^)	23.2 ± 3.6	23.1 ± 3.4	.952
Hemoglobin (g/dL)	8.8 ± 1.6	8.0 ± 1.6	<.001
Calcium (mmHg)	7.7 ± 1.0	7.4 ± 1.2	.001
Phosphorus (mmHg)	5.5 ± 2.0	6.4 ± 2.2	<.001
Uric acid (mmHg)	8.1 ± 2.8	8.6 ± 2.6	.010
Total cholesterol (mmHg)	157.2 ± 50.7	156.1 ± 46.7	.801
hs-CRP at 0 m	5.6 ± 08.8	4.3 ± 15.5	.383
hs-CRP at 3 m	2.7 ± 12.9	1.5 ± 5.9	.056

TCVC: tunneled central venous catheter; SD: standard deviation; NTCVC: non-tunneled central venous catheter; ESRD: end stage renal disease; DM: diabetes mellitus; MCCI: modified Charlson comorbidity index; CAD: coronary artery disease; CHF: congestive heart failure; CVD: cerebrovascular disease; PVD: peripheral vascular disease; SBP: systolic blood pressure; DBP: diastolic blood pressure; BMI: body mass index; hs-CRP: highly sensitive C-reactive protein.

### Patients’ characteristics according to the types of permanent vascular access

The types of permanent vascular accesses were traced in a total of 1113 patients (Table 3). There were 914 (82.1%) patients with AVF and 199 (17.9%) with AVG. Among the 1071 patients who started HD via CVC, 721 patients changed to AVF and 155 patients changed to AVG within following one year. The rest of patients were either still on the CVC or lost to follow-up or died. Those in AVF group were more likely to be younger and have lower MCCI score which indicate fewer comorbid conditions compared with AVG group. Especially, the prevalence of DM was prominently higher in AVG group compared to AVF group. The prevalence of macrovascular diseases including CVD and PVD were also higher in AVG group.

**Table 3.** Baseline characteristics according to types of permanent vascular access.

	AVF *n*= 914 (82.1%) Mean ± SD or *n* (%)	AVG *n*= 199 (17.9%) Mean ± SD or *n* (%)	*p*
Age (years)	56.8 ± 13.5	61.7 ± 12.5	<.001
Sex, male	455 (64.7%)	87 (56.9%)	.068
Time referral (months)	48.7 ± 91.8	35.6 ± 45.6	.097
Type of referral			
Early referral	430 (64.7%)	84 (59.2%)	.215
Late referral	235 (35.3%)	58 (40.8%)	
Cause of ESRD			
DM	421 (59.9%)	109 (71.2%)	.075
Hypertension	91 (12.9%)	15 (9.8%)	
Glomerulonephritis	108 (15.4%)	15 (9.8%)	
Others	75 (10.7%)	11 (7.2%)	
Unknown	8 (1.1%)	3 (2.0%)	
Score of MCCI	5.2 ± 2.2	6.3 ± 2.4	<.001
Comorbidity			
DM	458 (65.3%)	118 (77.1%)	.005
CAD	90 (12.9%)	23 (15.0%)	.492
CHF	71 (10.2%)	23 (15.0%)	.086
CVD	57 (8.2%)	22 (14.4%)	.018
PVD	55 (7.9%)	20 (13.1%)	.041
Malignancy	50 (7.2%)	18 (11.8%)	.059
SBP (mmHg)	144.4 ± 22.9	144.0 ± 21.3	.839
DBP (mmHg)	78.2 ± 14.2	73.5 ± 12.8	<.001
BMI (kg/m^2^)	23.3 ± 3.6	23.2 ± 3.6	.714
Hemoglobin (g/dL)	8.8 ± 1.7	8.9 ± 1.5	.704
Calcium (mg/dL)	7.7 ± 1.1	7.8 ± 0.91	.343
Phosphorus (mg/dL)	5.7 ± 2.0	5.4 ± 1.8	.129
Uric acid (mg/dL)	8.2 ± 2.5	7.9 ± 2.5	.195
Total cholesterol (mg/dL)	156.0 ± 47.9	155.4 ± 53.4	.896
hs-CRP 0 m	2.7 ± 11.8	3.9 ± 20.1	.331

AVF: arteriovenous fistula; SD: standard deviation; AVG: arteriovenous graft; ESRD: end stage renal disease; DM: diabetes mellitus; MCCI: modified Charlson comorbidity index; CAD: coronary artery disease; CHF: congestive heart failure; CVD: cerebrovascular disease; PVD: peripheral vascular disease; SBP: systolic blood pressure; DBP: diastolic blood pressure; BMI: body mass index; hs-CRP: highly sensitive C-reactive protein.

### Patients’ survival according to the type of initial and permanent vascular access

**Figure 1.**
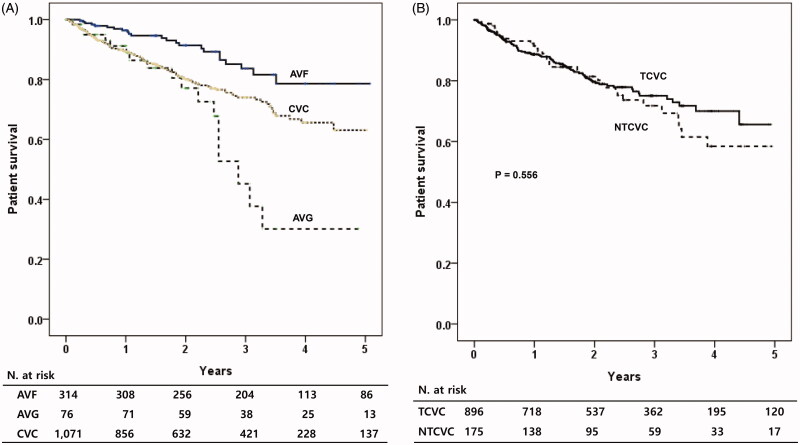
Patients’ survival according to the types of initial vascular access. (A) Comparisons among the AVF, AVG and CVC patients. (B) Comparison between NTCVC and TCVC. AVF: arteriovenous fistula; AVG: arteriovenous graft; CVC: central venous catheter; NTCVC: non-tunneled CVC; TCVC: tunneled CVC.

**Figure 2.**
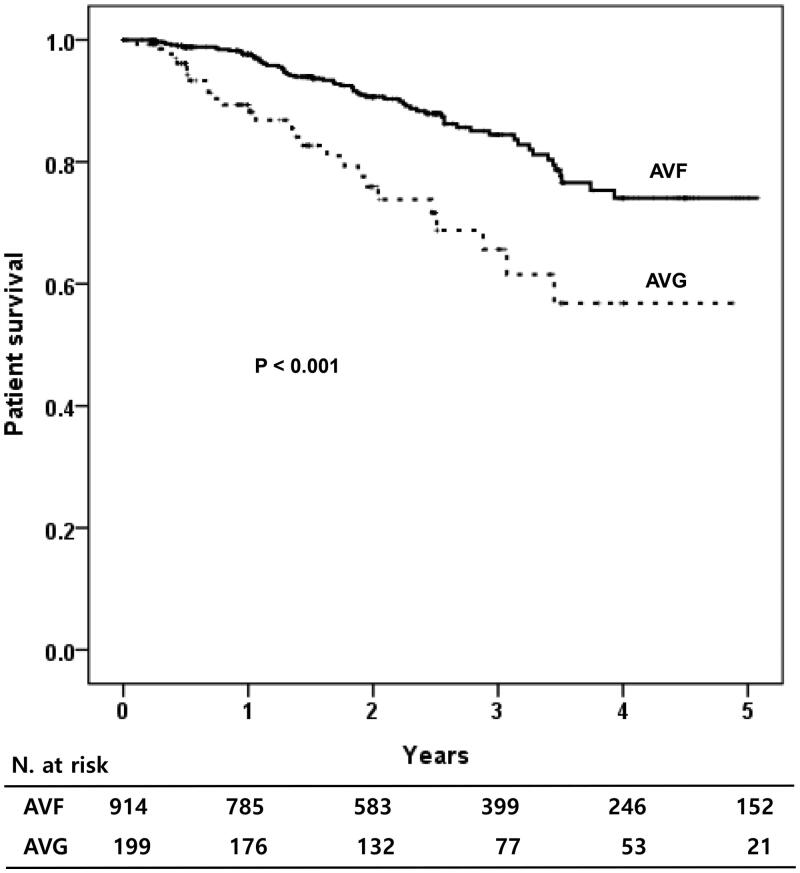
Patients’ survival according to the types of permanent vascular access. AVF: arteriovenous fistula; AVG: arteriovenous graft.

During 1.9 years of mean follow-up period, 182 patients (12.5%) were died. When the patients’ survival was compared according to the types of initial vascular access, patients with AVF showed significantly better survival compared to those with other vascular accesses (*p* < .001) (Figure 1(A)). AVG group showed the worst patients’ survival among the three types of vascular accesses. When the TCVC and NTCVC groups were compared, the patient survival was similar between the two groups (Figure 1(B)). When the patients’ survival was analyzed according to the types of permanent vascular access, the patients having permanent AVF showed significantly better survival rate compared to those with AVG (*p* < .001, Figure 2).

### Comparisons of HRQOL and BDI scores according to the type of initial vascular access

Table 4 shows the comparisons of HRQOL and BDI scores according to the type of initial vascular access at 3 months after beginning of HD. On comparing the KDQOL-36 according to the vascular access type, the AVF group showed significantly higher scores in seven of 10 SF-36 domains and the AVG group showed higher scores in two of 10 SF-36 domains than did the CVC group. The AVF group showed higher scores in three of 12 kidney disease targeted domains and the AVG group showed higher scores in two of 12 kidney disease targeted domains than did the CVC group. Even after adjusting for confounding variables including age, sex, type of referral, MCCI and hemoglobin level, the AVF and AVG group showed higher scores in six domains (physical composite summary, mental composite summary, physical functioning, role-physical, role-emotional, and social functioning) and two domains (mental composite summary and role-emotional) in SF-36 domains, respectively. After adjusting for confounding variables, the AVF and AVG group showed higher scores in six domains (symptoms/problems, effects of kidney disease, cognitive functioning, quality of social interaction, social support, and overall health) and two domains (symptoms/problems and patient satisfaction) in kidney disease targeted domains, respectively. BDI score was also significantly lower in AVF group which indicate less depressive mood after adjusting multiple variables (*p* = .024).

**Table 4.** Health-related quality of life (HRQOL) and Beck’s depression inventory (BDI) scores at 3 months after dialysis beginning according to the types of initial vascular access.

	AVF (*n*= 215) Mean ± SD	AVG (*n*= 48) Mean ± SD	CVC (*n*= 608) Mean ± SD	*p*^†^	*p*^‡^
HRQOL measure					
SF-36					
PCS	42.1 ± 9.3	38.7 ± 8.5	39.6 ± 9.5	.021*	.024*
MCS	42.6 ± 9.3	43.9 ± 7.6	39.7 ± 9.9	.002*^,^**	.005*^,^**
Physical functioning	73.6 ± 22.9	62.7 ± 26.9	65.9 ± 27.4	.010*	.008*
Role-physical	49.1 ± 41.6	39.1 ± 41.1	38.1 ± 41.0	.022*	.015*
Bodily pain	71.4 ± 23.6	72.9 ± 23.0	66.2 ± 24.9	.047*	.108
General health	37.0 ± 19.4	32.2 ± 18.0	35.5 ± 18.0	.370	.114
Mental health	54.9 ± 15.6	55.1 ± 16.4	52.1 ± 18.1	.202	.124
Role-emotional	67.1 ± 42.8	74.0 ± 37.6	49.0 ± 44.9	<.001*^,^**	<.001*^,^**
Social functioning	68.6 ± 24.1	64.8 ± 26.1	61.7 ± 25.9	.019*	.008*
Vitality	45.5 ± 18.2	44.7 ± 16.6	42.6 ± 19.9	.292	.100
Kidney disease targeted					
Symptoms/problems	82.4 ± 14.1	83.1 ± 12.6	78.1 ± 16.6	.008*^,^**	.016*^,^**
Effects of kidney disease	71.5 ± 16.3	69.6 ± 16.5	67.2 ± 19.5	.057	.018*
Burden of kidney disease	33.2 ± 21.6	34.6 ± 18.6	30.7 ± 22.6	.376	.413
Work status	27.1 ± 35.2	28.1 ± 28.2	23.5 ± 31.9	.429	.622
Cognitive functioning	87.1 ± 15.1	84.8 ± 14.8	81.1 ± 20.7	.005*	.002*
Quality of social interaction	69.5 ± 19.3	69.2 ± 17.7	63.4 ± 21.1	.005*	.004*
Sexual function	76.5 ± 25.9	66.7 ± 33.2	68.5 ± 34.3	.381	.145
Sleep	67.8 ± 19.8	71.0 ± 23.3	68.1 ± 19.4	.711	.97
Social support	57.3 ± 25.8	59.4 ± 23.9	62.0 ± 24.3	.144	.011*
Dialysis staff encouragement	85.3 ± 19.1	90.6 ± 13.5	84.3 ± 19.1	.187	.618
Overall health	55.6 ± 18.7	54.7 ± 16.1	51.3 ± 19.6	.061	.024*
Patient satisfaction	68.8 ± 23.1	76.6 ± 20.7	65.9 ± 22.6	.024**	.014**
Mean	60.6 ± 17.3	59.8 ± 18.2	56.1 ± 16.9		
BDI	13.8 ± 9.5	13.7 ± 8.0	16.6 ± 11.0	.020*	.024*

AVF: arteriovenous fistula; SD: standard deviation; AVG: arteriovenous graft; CVC: central venous catheter; HRQOL: health-related quality of life; SF-36: short form 36; PCS: physical composite summary; MCS: mental composite summary; BDI: Beck’s depression inventory.

^†^ Value was obtained from unadjusted regression analysis.

^‡^ Value was obtained from regression analysis adjusted for age, sex, type of referral, modified Charlson comorbidity index, and hemoglobin levels.

^*^ AVF versus CVC.

^**^ AVG versus CVC.

At 1 year after beginning of HD, almost all the KDQOL-36 scores slightly increased compared to those at 3 months after dialysis beginning (Table 5), and the average score increased from 60.6 to 62.5 in AVF group, 59.8–62.4 in AVG group and 56.1–56.6 in CVC group. The AVF and AVG group had higher mean scores in each SF-36 domain than did the CVC group, and this difference was statistically significant in five domains and one domain, respectively. After adjusting for confounding variables, physical composite summary, role-physical, bodily pain, and role-emotional domain were significantly higher in AVF group. AVG group had higher score in role-emotional domain (*p* = .017). In adjusted analyses of kidney disease targeted domains, AVF group showed significantly higher scores in two (symptoms/problems and patient satisfaction) and the AVG group showed significantly higher scores in two (overall health and patient satisfaction) of 12 domains than did the CVC group. Mean BDI scores were 12.9 and 17.3 in AVF and CVC groups, respectively. The difference was significant before (*p* = .003) and after adjustment (*p* = .009), and this implies that patients with AVF are also less depressive at 1 year after beginning of dialysis.

**Table 5.** Health-related quality of life (HRQOL) and Beck's depression inventory (BDI) scores at 12 months after dialysis beginning according to the types of initial vascular access.

	AVF (*n*= 186) Mean ± SD	AVG (*n*= 48) Mean ± SD	CVC (*n*= 394) Mean ± SD	*p*^†^	*p*^‡^
HRQOL measure					
** **SF-36					
** **PCS	45.2 ± 9.5	43.9 ± 10.1	41.5 ± 9.2	.006*	.008*
** **MCS	42.8 ± 8.7	42.1 ± 8.0	40.3 ± 9.1	.080	.145
** **Physical functioning	72.6 ± 21.9	70.9 ± 22.1	65.5 ± 25.9	.066	.129
** **Role-physical	74.4 ± 32.4	67.4 ± 31.9	57.2 ± 36.0	<.001*	.004*
** **Bodily pain	74.6 ± 21.0	73.2 ± 24.1	65.8 ± 22.4	.005*	.006*
** **General health	49.6 ± 21.6	48.9 ± 20.1	45.7 ± 21.7	.324	.233
** **Mental health	53.9 ± 16.8	54.6 ± 17.3	50.9 ± 17.1	.302	.567
** **Role-emotional	72.2 ± 40.1	73.9 ± 37.6	55.2 ± 42.1	.002*^,^**	.017*^,^**
** **Social functioning	67.8 ± 30.2	65.7 ± 31.3	58.0 ± 32.5	.045*	.065
** **Vitality	54.8 ± 14.2	50.9 ± 15.1	50.7 ± 17.3	.145	.258
** **Kidney disease targeted					
** **Symptoms/problems	85.3 ± 12.1	86.2 ± 13.3	79.7 ± 16.3	.005*	.013*
** **Effects of kidney disease	70.0 ± 16.8	71.6 ± 17.8	69.6 ± 19.7	.880	.872
** **Burden of kidney disease	31.0 ± 20.0	34.0 ± 26.7	30.3 ± 22.9	.757	.78
** **Work status	42.0 ± 23.5	34.0 ± 67.7	31.3 ± 23.2	.234	.945
** **Cognitive functioning	69.0 ± 17.4	67.8 ± 11.3	64.8 ± 18.8	.181	.164
** **Quality of social interaction	70.0 ± 19.5	73.8 ± 18.5	64.5 ± 20.4	.022*	.084
** **Sexual function	67.1 ± 25.8	72.9 ± 30.0	68.2 ± 32.7	.914	.866
** **Sleep	66.0 ± 21.1	65.1 ± 25.7	59.9 ± 22.5	.080	.101
** **Social support	73.6 ± 16.0	71.6 ± 20.6	69.0 ± 20.6	.162	.151
** **Dialysis staff encouragement	76.6 ± 15.0	81.7 ± 16.4	74.9 ± 18.6	.185	.375
** **Overall health	57.8 ± 48.8	60.9 ± 49.9	49.4 ± 45.7	.007**	.004**
** **Patient satisfaction	59.1 ± 17.9	60.9 ± 19.1	53.3 ± 20.1	.025**	.031*^,^**
** **Mean	62.5 ± 13.8	62.4 ± 14.6	56.6 ± 13.3		
BDI	12.9 ± 9.4	14.0 ± 8.5	17.3 ± 10.8	.003*	.009*

AVF: arteriovenous fistula; SD: standard deviation; AVG: arteriovenous graft; CVC: central venous catheter; HRQOL: health-related quality of life; SF-36: short form 36; PCS: physical composite summary; MCS: mental composite summary; BDI: Beck’s depression inventory.

^†^ Value was obtained from unadjusted regression analysis.

^‡^ Value was obtained from regression analysis adjusted for age, sex, type of referral, modified Charlson comorbidity index, and hemoglobin levels.

^*^ AVF versus CVC.

^**^ AVG versus CVC.

### Frequency of hospitalization

The frequencies of annual hospitalization according to the type of vascular access are shown in Table 6. Regarding the four types of initial vascular access, patients with NTCVC tended to be hospitalized more frequently, but it was not statistically significant. However, the frequency of hospitalization was significantly different according to the permanent vascular access, which was higher in patients with AVG compared to those with AVF (AVF 0.7 vs. AVG 1.1 times per year) (*p* = .024).

**Table 6.** Frequency of annual hospitalization according to the types of vascular access.

Type of vascular access	Annual hospitalization Mean ± SD	*p*
Initial access		
AVF	0.6 ± 1.3	.092
AVG	0.7 ± 1.4	
TCVC	0.6 ± 1.4	
NTCVC	0.9 ± 2.0	
Permanent access		
AVF	0.7 ± 1.5	.024
AVG	1.1 ± 1.7	

SD: standard deviation; AVF: arteriovenous fistula; AVG: arteriovenous graft; TCVC: tunneled central venous catheter; NTCVC: non-tunneled central venous catheter.

## Discussion

In this multi-center prospective cohort study, we analyzed the all-cause mortality, HRQOL, and BDI scores according to the type of vascular access in incident HD patients. Similar to previous studies [20,21], the survival rate of patients with AVF was higher than those of patients with AVG or CVC. Furthermore, the HRQOL score was high and the BDI score was low in patients with AVF or AVG compared to those with CVC both 3 months and 1 year after the beginning of dialysis. These data suggest that the types of vascular access at the initiation of HD affect the physical and mental health as well as survival rate.

The patients who started dialysis with AVG were older and had more comorbidity compared to those with AVF or CVC. And also, the patients who had AVG as a permanent vascular access had higher prevalence of DM and macrovascular complications. As we expected, the survival rate was worst in patients with AVG probably due to the severe comorbidity and older age. There was no significant difference in survival rate between tunneled and NTCVC as an initial vascular access. The risk of hospitalization seemed to be higher in patients with NTCVC, but it was not statistically significant. The patients with AVG as a permanent vascular access suffered frequent hospitalization compared to AVF patients. Although we could not discriminate the exact causes of hospitalization, the patients with AVG hospitalized frequently probably due to macrovascular diseases and infectious complications led by multiple risk factors [1,21,22].

The AVF is recognized as the preferred type of vascular access for its longer patency, fewer infectious complications, and associated with lower all-cause mortality compared with AVG or CVC [1–3]. However, only a few studies have examined the relationship between the physical or mental health and vascular access type. Recently, in a small study, Domenick Sridharan et al. showed that HD patients experienced great satisfaction with AVF, but there was no significant independent association of vascular access type with HRQOL [12]. In another single center study, Afsar et al. assessed the HRQOL and depression according to the vascular access type in patients receiving HD [10]. They suggested that having a CVC influence negatively in HRQOL. Wasse et al. revealed that compared with persistent CVC use, early permanent AVF use was associated with the perception of improved health status and quality of life among patients with ESRD [11]. In this study, we used the KDQOL-36 survey, which contains kidney disease-specific questions. It showed similar results to those of previous studies in terms of HRQOL. The patients with AVF or AVG showed higher physical functioning and social functioning scores and were more likely to be satisfied with their life than patients with CVC. Moreover, this study showed that the score of HRQOL improved as the vintage of dialysis increased from 3 months to 1 year.

In the study, we also analyzed the patients’ depression score according to the type of vascular access. Weisbord et al. reported that physical and emotional symptoms were common and severe in HD patients, and were correlated directly with impaired HRQOL and depression [23]. In previous study, vascular access type was not associated with the depression [10]. However, our study showed that the degree of depression was related to the vascular access type. The patients with CVC had significantly higher BDI score compared with those with AVF or AVG. Contradictory to previous studies, this study was performed prospectively and included relatively large number of incident HD patients. It showed clearly that AVF had beneficial effect regarding patients’ mood. To the best of our knowledge, this is the only large prospective study that comprehensively elucidated the difference of BDI scores according to vascular access type. This finding might reflect the increased dialysis adequacy by AVF or AVG use which leads to greater reduction of uremic toxins and better anemia correction compared with CVC use [24,25]. Also, there is a possibility that the presence of CVC in upper body incurs disturbed body image and depression. In patients who are not candidate for the creation of AVF which is the best option in HD patients in terms of QOL and depression, every effort is needed to enhance recognition of annoying symptoms such as depression and pain and to improve the effects of intervention [26].

There were significant differences in the context of referral time according to the initial vascular access. The referral time was relatively long before the start of HD and the proportion of patients with early referral was high in patients having AVF as an initial access. However, more than half of patients referred to nephrologists ≥1 year before the initiation of dialysis started HD via temporary CVC. It implies that there is still much opportunity to increase the proportion of patients who create permanent vascular access long before the start of dialysis and avoid the use of CVC. Fortunately, the proportion of AVF as a permanent vascular access in this study was greater than 80%, which was significantly high compared to those of other countries [1,3]. South Korea is one of the highly prevalent countries in the world regarding ESRD. Therefore, early creation and regular surveillance of permanent vascular access is an important task for the care of ESRD patients [27].

Although the results of this study are informative in terms of HRQOL and depression linked to the vascular access type, this study has several limitations. First, only 871 patients at 3 months and 628 patients at 12 months after initiation of dialysis completed HRQOL and BDI surveys. Therefore, there might be a possibility of selection bias. At least, when we compared the clinical characteristics of patients according to the participation status of surveys, there were no significant differences between patients who participated and those who did not. Second, the number of patients started HD via AVG was relatively small compared with AVF or CVC, and it was difficult to make an accurate comparison. In addition, we could not follow the whole enrolled patients and the total number of patients that we could determine the permanent vascular access was less than expected nonetheless considering the censored patients due to early death. Despite of these limitations, we confirmed the importance of initial HD vascular access type in a large prospective cohort study that included only incident patients, and comprehensively elucidated the difference of HRQOL and BDI scores according to the vascular access type.

In conclusion, the survival rate of patients with AVF was higher than those of patients with AVG or CVC. Also, the HRQOL score was high and the BDI score was low in patients with AVF or AVG compared to those with CVC both 3 months and 1 year after the beginning of dialysis. These data suggest that the types of vascular access at the initiation of HD affect the physical and mental health as well as the survival rate. Therefore, it might be essential for the care of impending ESRD patients to prepare permanent vascular access preemptively and avoid the use of CVC as an initial vascular access.
